# Peatland Ecosystem Processes in the Maritime Antarctic During Warm Climates

**DOI:** 10.1038/s41598-017-12479-0

**Published:** 2017-09-27

**Authors:** Julie Loisel, Zicheng Yu, David W. Beilman, Karl Kaiser, Ivan Parnikoza

**Affiliations:** 1 Department of Geography, Texas A&M University, College Station, USA; 2 Department of Earth and Environmental Sciences, Lehigh University, Bethlehem, USA; 3 Department of Geography, University of Hawai‘i at Mānoa, Honolulu, USA; 4 Department of Marine Sciences, Texas A&M University, Galveston, USA; 5 Institute of Molecular Biology and Genetics, National Academy of Sciences, Kyiv, Ukraine

## Abstract

We discovered a 50-cm-thick peat deposit near Cape Rasmussen (65.2°S), in the maritime Antarctic. To our knowledge, while aerobic ‘moss banks’ have often been examined, waterlogged ‘peatlands’ have never been described in this region before. The waterlogged system is approximately 100 m^2^, with a shallow water table. Surface vegetation is dominated by *Warnstorfia fontinaliopsis*, a wet-adapted moss commonly found in the Antarctic Peninsula. Peat inception was dated at 2750 cal. BP and was followed by relatively rapid peat accumulation (~0.1 cm/year) until 2150 cal. BP. Our multi-proxy analysis then shows a 2000-year-long stratigraphic hiatus as well as the recent resurgence of peat accumulation, sometime after 1950 AD. The existence of a thriving peatland at 2700–2150 cal. BP implies regionally warm summer conditions extending beyond the mid-Holocene; this finding is corroborated by many regional records showing moss bank initiation and decreased sea ice extent during this time period. Recent peatland recovery at the study site (<50 years ago) might have been triggered by ongoing rapid warming, as the area is experiencing climatic conditions approaching those found on milder, peatland-rich sub-Antarctic islands (50–60°S). Assuming that colonization opportunities and stabilization mechanisms would allow peat to persist in Antarctica, our results suggest that longer and warmer growing seasons in the maritime Antarctic region may promote a more peatland-rich landscape in the future.

## Introduction

**Figure 1:**
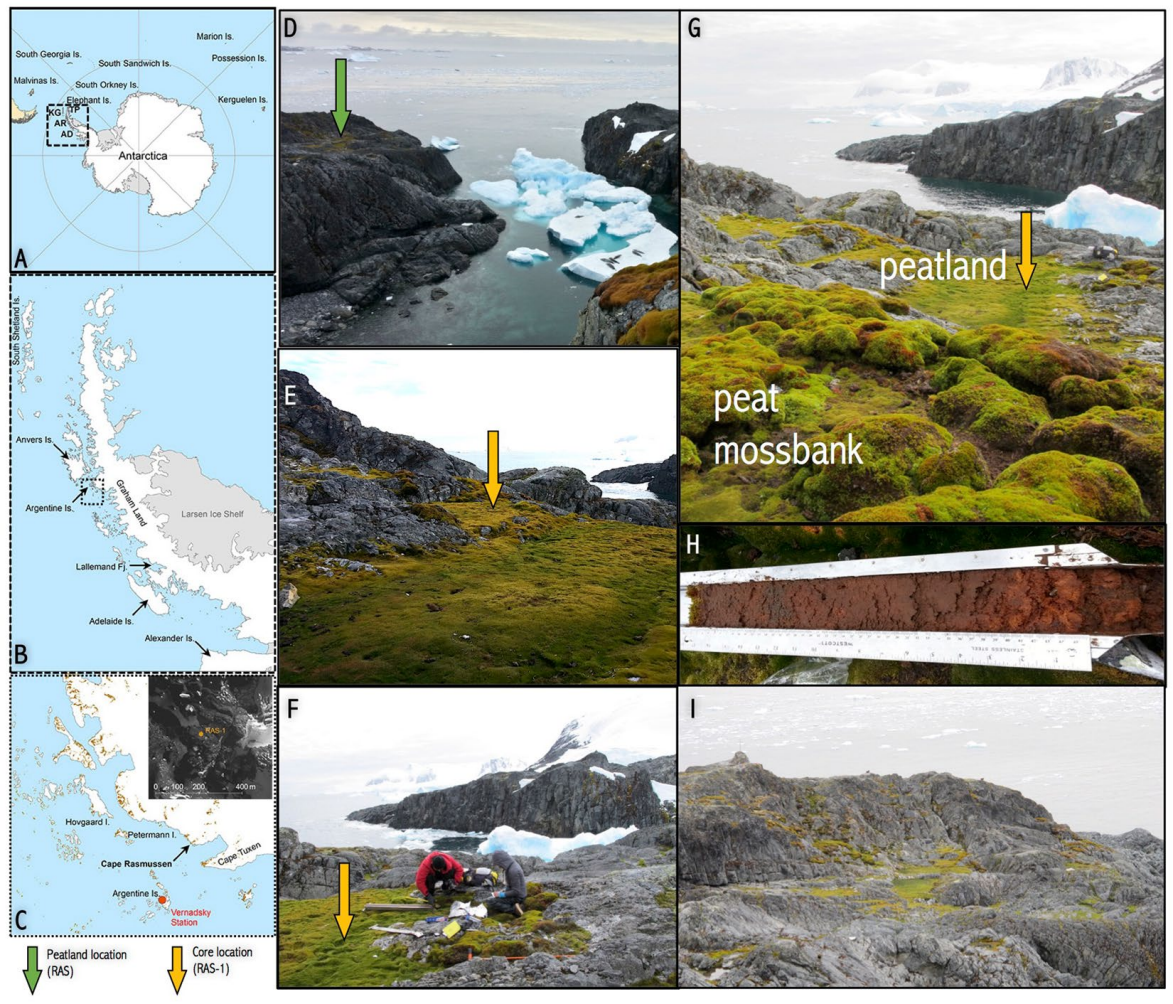
A surprisingly green and lush maritime Antarctic landscape at Cape Rasmussen, Graham Coast (65°S, 64°W). Location maps of Antarctica (**A**), the Antarctic Peninsula and locations discussed in the text (**B**), and the study area (**C**). The study peatland (RAS) is shown from several angles (**D**–**G**); panel G contrasts the peculiar low-lying, waterlogged peatland depression described in this study to the aerobic moss banks typically found across the landscape, often on rocky slopes. Core RAS-1 was used to reconstruct the peatland’s paleoenvironmental history (**H**). Overview of the rocky, yet relatively lush, Graham Coast in proximity to Cape Rasmussen (**I**). KG: King George Island; TP: Trinity Peninsula; AR: Argentine Islands; AD: Adelaide Island. Maps created by D.B. at the University of Hawaiʻi at Mānoa (Dept. of Geography) using ArcGIS v10.2 (http://www.arcgis.com). Photo credit: DB, JL, ZY.

The maritime Antarctic biogeographic region has been experiencing one of the most rapid rates of warming worldwide in recent decades^1^. Reductions in snow and ice cover^2^, retreat of glaciers^3,4^, the southward range expansion of the only two Antarctic flowering plant species^5^, as well as increases in moss bank (also called ‘moss peatbanks’ in the literature) accretion rates^6^ have all been linked to recent warming. Here we report the recent resurgence of a peatland on Cape Rasmussen (65.247°S, 64.085°W), off the Graham Coast of the Antarctic Peninsula (Fig. 1). To our knowledge, ‘peatlands’ — that is, saturated peat-forming ecosystems with dynamic but persistent near-surface water tables — have never been reported in Antarctica, although two buried peat sequences have been described^7,8^ and many aerobic ‘moss banks’ have been examined^6^. We interpret peatland initiation, around 2700 calibrated years before present (cal. BP), as well as its recent resurgence, less than 50 years ago, as new and important indicators of ecosystem changes that have responded to warmer conditions in the region.

In the maritime Antarctic region, locally abundant plant cover is dominated by at least 125 bryophytes and 250 lichens species^9^. The region also supports two vascular plants species, *Deschampsia antarctica* E. Desv. and *Colobanthus quitensis* (Kunth) Bartl., which are sparsely distributed across the ice-free land area^10^. Many of the wet-adapted moss species—including *Sanionia georgicouncinata* (Müll. Hal.) Ochyra & Hedenäs*, Sanionia uncinata* (Hedw.) Loeske*, Brachythecium austrosalebrosum* (Mäll.Hal.) Kindb*., and Warnstorfia fontinaliopsis* (Müll. Hal.) Ochyra—form thin carpets that can reach up to 5–10 cm in thickness^7^; they act as colonizing species in depressions and cracks on barren rocks. On the other hand, mosses *Polytrichum strictum* Brid. and *Chorisodontium aciphyllum* (Hook. f. & Wilson) Broth. are typically found in drier areas, where they tend to form turves and, at times, accrete over hundreds or even thousands of years into thick deposits (up to 3 m) commonly referred to as ‘moss banks’. These moss banks have been abundantly described in the literature over the past few decades^11^, and a few of them have been used as natural archives to reconstruct past environmental changes^6,8,12^. Noteworthy is that moss banks are aerobic, lacking a water table, and are therefore not considered wetlands; the thickest banks also tend to support permafrost in their deepest layers. In contrast, the reported peat deposit on Cape Rasmussen conforms to the ‘peatland’ definition: it is characterized by a peat deposit in excess of 40 cm in depth, its soil is saturated, it supports a prevalence of hydrophytic vegetation, and its peat layer consists of at least 80% (dry mass) of dead organic material. In fact, the similarity between the Cape Rasmussen peatland and fens from the boreal and subarctic regions is striking in terms of their visual appearance, location in the landscape (wet depressions), plant structure (moss-dominated), and carbon sequestration function, making the rarity of peatlands in the maritime Antarctic region intriguing.

## Cape Rasmussen peatland: a unique ecosystem in Antarctica

**Figure 2:**
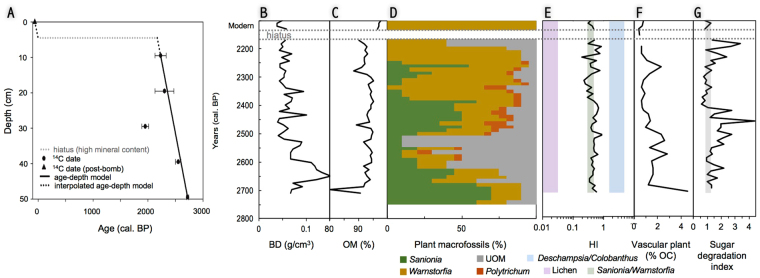
Stratigraphic analysis (core RAS-1) reveals the inception and rapid development of a peatland ecosystem in Antarctica around 2700 cal. BP. The age-depth model indicates high accumulation rates (0.1 cm/yr) between 2700 and 2150 cal. BP, followed by a long hiatus in peatland development and recent resurgence (**A**). Peat physical properties (bulk density (BD) and organic matter (OM) content) are reportedly similar to those found in functionally comparable peatland ecosystems across the globe (**B** and **C**). Plant macrofossil analysis shows that *Sanionia* sp. colonized the site and was eventually succeeded by *Warnstorfia fontinaliopsis*; UOM: unidentified organic matter (**D**). The hemicellulosic index (HI) independently confirms the dominance of *Sanionia* and *Warnstorfia* throughout the sequence; vertical bands show HI values for various vegetation groups (**E**). Lignin phenols show that a small percentage of the total organic carbon (OC %) comes from vascular plants *Deschampsia* and/or *Colobanthus* (**F**). The sugar degradation index shows periods of intensified decay centered around 2625, 2500, and 2200 cal. BP (**G**).

The Cape Rasmussen peatland (RAS) covers approximately 100 m^2^ and is characterized by a shallow water table (7 cm), which was measured on 21 February 2014 (Fig. 1). Surface vegetation is dominated by *Warnstorfia fontinaliopsis*, a wet-adapted moss species found along the west Antarctic Peninsula. We observed a few other rocky depressions nearby that were infilled by moss, but we did not sample them for conservation reasons. The studied core (RAS-1) is 50 cm in length; it has a high organic matter content (>90% by weight) and bulk density averages 0.067 g/cm^3^ (Fig. 2). These values resemble those reported for peatlands globally^13,14^. The stratigraphic chronology was constrained by six radiocarbon (^14^C) dates (Fig. 2, Table S1). The visible stratigraphic sequence is almost exclusively composed of moss remnants, with the exception of a sand-rich horizon at 4–6 cm below the surface, which we interpret as a disturbed surface (Fig. 2). From the time of inception at 2700 cal. BP until approximately 2150 cal. BP, a steady yet relatively rapid mean peat accumulation rate was reconstructed (~0.1 cm/year). Peat accumulation seems to have been interrupted from 2150 cal. BP until at least 1950 AD, as evidenced by a stratigraphic hiatus (Fig. 2). The uppermost 1-cm peat horizon returned a modern radiocarbon age (‘post-bomb’, i.e., younger than 1950 AD) and is composed of live *Warnstorfia fontinaliopsis*.

Plant macrofossil analysis reveals a potential hydroseral pathway for peatland development in Antarctica (Fig. 2). The site was first colonized by carpet-forming *Sanionia* sp. around 2700 cal. BP. *Warnstorfia fontinaliopsis* subsequently co-dominated the community assemblage with *Sanionia* for approximately 300 years, until it became the dominant species at 2400 cal. BP. The presence of *Polytrichum strictum* from 2500 to 2300 cal. BP may indicate episodic surface drying^8^. Compositions of hemicellulosic carbohydrates, an emerging biochemical indicator^15^, independently show the dominance of moss remains throughout the core (Fig. 2). Minor but consistent contributions of *Deschampsia* and/or *Colobanthus* remains (<5% of total organic carbon) are indicated by the presence of vanillyl and syringyl phenols, which are only synthesized by vascular plants^16^.

Peat decomposition varied along the core, as shown by a suite of additional biochemical markers (Fig. 2). Our sugar degradation index, which was calculated as the ratio of the expected sugar yield to the observed yield, shows spikes in peat decomposition centered around 2625, 2500, and 2200 cal. BP (Fig. 2). Enhanced decomposition in these layers may either be linked to surface drying associated with extended oxygen exposure time, or to the presence of more labile material such as *Sanionia* or *Deschampsia*/*Colobanthus* remnants. The zone of extensive decomposition between 2550 and 2425 cal. BP overlaps with the abundant occurrence of unidentified organic matter (UOM) and the presence of *Polytrichum strictum*, two indicators of surface drying. The decomposed intervals around 2625 and 2200 cal. BP are both synchronous with high UOM percentages. On the basis of these findings, we confirm that oxygen exposure time and vegetation types are important controls on decomposition in Antarctica, as they are in arctic peatlands^17^.

The sharp contrast from *Warnstorfia*-dominated peat to mineral-rich sediments at 6.5 cm suggests a disruption in sediment accumulation. The inorganic layer is mostly composed of sand-sized mineral grains (65%) and unidentifiable organic matter (35%); a few *Warnstorfia* leaves were also identified within the matrix. Above it, from 4.5 cm to the surface, live *Warnstorfia* mosses have recently recolonized the site. On the basis of radiocarbon dating (Table S1), the inorganic horizon from 6 to 4 cm below the surface is interpreted as representing a hiatus in peatland development from 2150 cal. BP until ~1950 AD. This stratigraphic information alone is insufficient to infer whether peat accumulation was interrupted for approximately 2000 years or if some peat was eroded from the record at some time between these dates. That said, combining this information with other local paleo records allows us to speculate that Cape Rasmussen might have been covered by permanent snow or ice sometime in the past 2000 years. Cold conditions during the late Holocene (approximately 900 to 100 cal. BP) have been reconstructed from multiple sites across the region and linked to glacial advance^27,31^ as well as a decline in moss bank inception rate^8^ along the Graham Coast and neighboring islands. Likewise, an abundance of kill ages from ice-entombed mosses recently exposed from retreating glacial age near Palmer and Vernadsky stations suggest cold conditions during the period from 900–600 cal. BP^8^. In addition, a moss bank core (RAS-2), which was collected uphill and less than 500 m away from the Cape Rasmussen peatland (RAS-1), similarly shows a stratigraphic hiatus from 1200 cal. BP to 1960 AD^8^. While the hiatus at RAS-2 is shorter than the one observed at RAS-1, these combined records point to an event that shut down peat accumulation locally. We argue that the observed mineral grains at both sites may be relics of a time where permanent snow or ice dominated Cape Rasmussen’s landscape. Glacial ice advance could have eroded some of the peat sequences, and/or transported the sand-sized grains to the coring sites. The difference in timing could be due to the erosion at RAS-1 (i.e., ‘missing peat’ from 2150 to 1200 cal. BP), and/or to an earlier onset of permanent snow/ice at the peatland site, which is low-lying as well as wind-protected (Fig. 1). Lastly, recent recolonization of the study site by *Warnstorfia* mosses is interpreted as a response to recent rapid warming that has been observed across the region^2–6^; it is synchronous with the establishment and rapid growth of *Polytrichum* moss bank at RAS-2^8^. That said, and given our unique observation during a single summer season, we do not know how stable the recolonized *Warnstorfia* moss ecosystem is.

Overall, the Cape Rasmussen peatland likely underwent a succession from a wet and thin *Sanionia* carpet to a waterlogged *Warnstorfia* peatland around 2700 cal. BP, with a period of intensified decomposition around 2500 cal. BP that probably corresponds to longer oxygen exposure time. The biochemical analysis further indicates the presence of *Deschampsia* and/or *Colobanthus*, which were completely decomposed downcore and only showed as biochemical signatures. The mineral-rich horizon, which is interpreted as representing a disruption in peatland development from 2150 cal. BP to approximately 1950 AD, was attributed to late-Holocene cooling conditions and possible permanent snow or ice coverage of our study site for some time between these two dates. Lastly, the recent recolonization of the study site by *Warnstorfia* mosses is interpreted as a response to regional recent rapid warming.

## Evidence for mild summer temperatures beyond the mid-Holocene in the maritime Antarctic region

**Figure 3:**
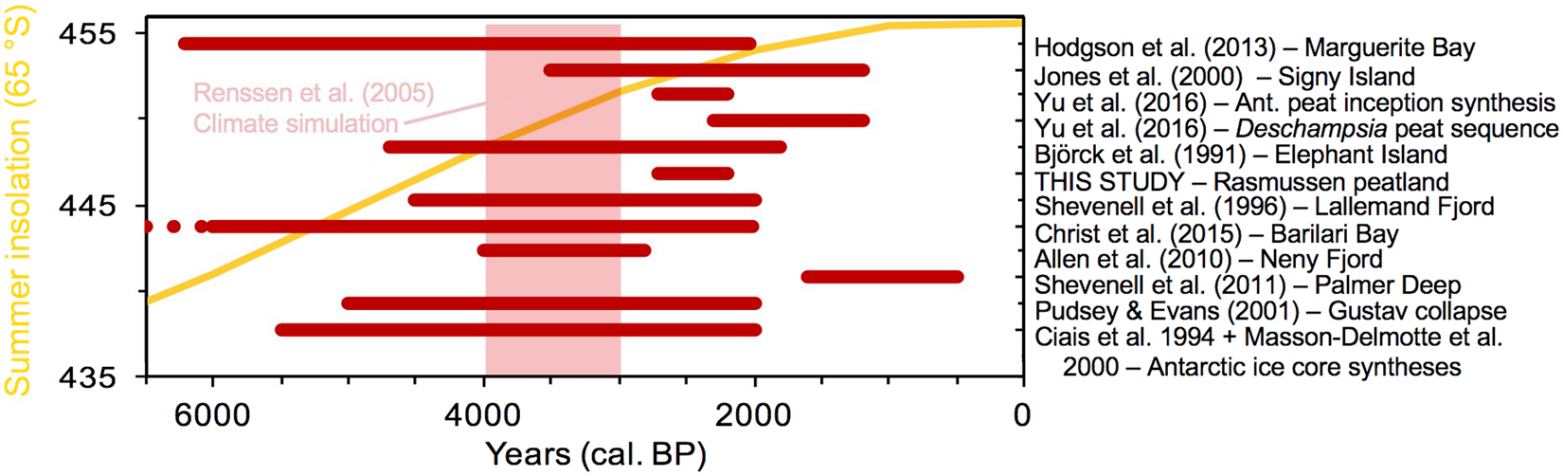
Compilation of mid- and late-Holocene climate change records along the Antarctic Peninsula. Changes in summer insolation at 65°S are presented in yellow (data from ref.^20^). A series of regional paleoenvironmental records from lakes, terrestrial ecosystems (including Cape Rasmussen peatland), marine sediments, and ice cores are presented; red horizontal bands represent time periods interpreted as mild by the original authors (details are provided in the text). The red dots indicate a warm period extending back to at least 7022 cal. BP^27^. The vertical pink band at 4000-3000 cal.BP shows the regional climate optimum as simluated by a model^21^. We note that, on multiple occasions, warm conditions persisted beyond the mid-Holocene thermal maximum.

The mid-Holocene has been broadly recognized as a period of climate amelioration along the Antarctic Peninsula^18^. Drivers for a mild mid-Holocene include a poleward shift of the southern westerly wind belt^19^ and a progressive increase in summer insolation^20^ (Fig. 3). A Holocene climate simulation for Antarctica supports an insolation-driven mild mid-Holocene, with a simulated climate optimum at 4000–3000 cal. BP and summer temperatures 1.3 °C warmer than present, followed by a cooling trend down to present-day values^21^.

Milder conditions during the mid-Holocene interval have been linked to several changes in lacustrine and terrestrial environments across the region (Fig. 3). For instance, rapid sedimentation and high organic productivity have been reported from a series of lakes from the maritime and sub-Antarctic regions, including South Georgia (54°S), Signy (60°S), the South Shetlands (62°S), and even the Marguerite Bay area (68°S)^22,23^. Most moss banks in maritime Antarctica initiated during the period between 2700 and 2200 cal. BP, which implies a climate suited for their development^8^. In addition, a fossilized peaty deposit composed of *Deschampsia antarctica*—a so-called *Deschampsia* bog that is only currently found in South Georgia (54°S)^24^—was discovered at the base of a modern-day dry moss bank sequence on Cape Rasmussen and dated at 2300–1200 cal. BP^8^. A moss bank from Elephant Island (61°S), in the maritime Antarctic region, recorded very high peat accumulation rates and high moss productivity from 4700 to 1800 cal. BP^25^. Lastly, a fossilized peaty layer dating back to approximately 5500 cal. BP was discovered on King George Island (62°S)^7^.

Marine records yield a more complex perspective of the mid-Holocene (Fig. 3), though most reconstructions also point to milder conditions. For instance, along the Graham Coast, marine cores from Lallemand Fjord (67°S) and Barilari Bay (65°S) indicate increased primary productivity, reduced sea-ice coverage, and greater meltwater-derived sedimentation during the mid-Holocene^26,27^. These events are synchronous with the breakup of Prince Gustav Channel Ice Shelf between 5000 and 2000 cal. BP, as recorded by ice-rafted debris (IRDs), which signals seasonally open water^28^. Likewise, compilations of ice core-based temperature reconstructions from the Antarctic continent generally point to warmer conditions from 4500 to 2000 cal. BP^29^ or from 5500 to 2500 cal. BP^30^. However, marine records that have emerged from Palmer Deep (ODP site 1098), off the coast of Anvers Island, indicate an abrupt and prolonged climate deterioration from 3600 cal. BP until present day^31^. Likewise, a subsequent study using TEX_86_ values suggests the occurrence of the lowest Holocene SSTs at 2700–1700 cal. BP, followed by warming at 1600–500 cal. BP^32^ (Fig. 3). A possible explanation for the discrepancy between SST reconstructions from Palmer Deep and previously mentioned records is a possible Spring response of pelagic marine archaea and thus of TEX_86_^32^ vs. a summer one for lacustrine, terrestrial, and IRD records. We know that austral spring (September-November) insolation has been decreasing throughout the Holocene, but that austral summer (December-February) has been increasing^20^. Winter upwelling inputs from the warm Circumpolar Deep Water and the Antarctic marine reservoir effect could also influence or complicate temperature reconstructions from Palmer Deep.

While the exact timing, duration, and intensity of the mild mid-Holocene period tend to differ between regions and type of archives, there is widespread evidence for its existence along the Antarctic Peninsula (Fig. 3). Some of these discrepancies may well be associated with seasonal proxy sensitivity, as discussed for the Palmer Deep record above^18^. At the local scale, multiple lines of evidence suggest an extended warm episode that ended during the late Holocene (Fig. 3). These include: (1) Cape Rasmussen peatland, with its initiation and subsequent rapid development between 2700 and 2150 cal. BP, (2) the fossilized peaty deposit of *Deschampsia antarctica* dated from 2300 to 1200 cal. BP, which points to mild summers^8^, (3) the inception of moss banks from 2700 to 2200 cal. BP^8^, and reduced sea-ice cover until 2000 cal. BP at Barilari Bay^27^. Lastly, should the stratigraphic hiatus at RAS-1 had been attributed to the erosion of a longer peat sequence, the latter would provide additional evidence of persistent warm conditions beyond the mid-Holocene thermal maximum.

## A greener Antarctica in the decades to come?

**Figure 4:**
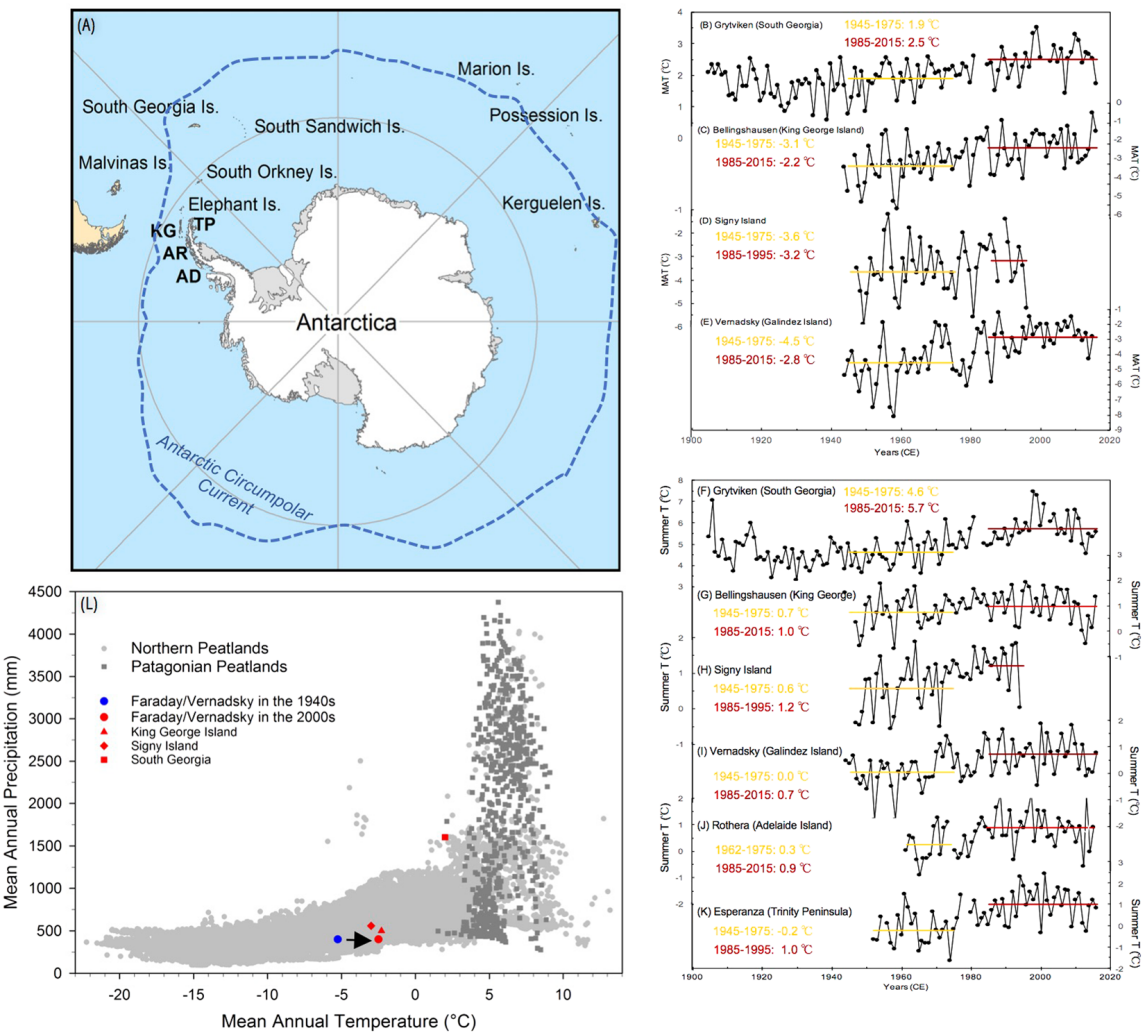
Recent temperature changes in the maritime Antarctic region analyzed in the context of peatland climate space. Location of sub-Antarctic and maritime Antarctic moss- and peat-bearing islands, and weather stations (**A**). Mean annual temperature (MAT) trends from South Georgia (54°S), Signy (60°S), King George (62°S), and Vernadsky (65°S) (**B–E**). Mean summer temperatures (DJF) for the same four stations as well as for Rothera (67°S) and Esperanza (60°S) (**F**–**K**). Global peatland climate space (**L**) showing the general distribution of northern^43^ and Patagonian^13^ peatlands, in addition to sub-Antarctic weather stations in proximity of moss banks and RAS peatland. KG: King George Island; TP: Trinity Peninsula; AR: Argentine Islands; AD: Adelaide Island. Map created by JL at Texas A&M University (Dept. of Geography) from ESRI data and maps using ArcGIS v10.3 (http://www.esri.com/data/data-maps).

Historical weather data from the maritime Antarctic region show rapid increasing trends of warming over the past few decades. A particularly strong rise in mean annual temperature has been recorded at the Faraday/Vernadsky station, at a rate of 5.7 °C per century^1^. While this rapid warming trend is mostly due to large increases in winter temperature, summer warming is significant and estimated at 2.4 °C per century^33^. The Antarctic summer season, defined here as the months of December, January, and February, is particularly important for terrestrial ecosystems who rely on short growing seasons that are often characterized by sub-zero temperatures. This is especially the case along the Antarctic Peninsula, as summer temperatures are close to freezing point (Fig. 4). A slight increase in the number of degree-days above freezing therefore presents a high potential for disruption of the cryosphere (melting) as well as an opportunity for organisms to grow longer and faster.

Every station along the Antarctic Peninsula shows an increase in summer temperature and a shift to a definitive positive degree-day climatic regime since ~1980 AD (Fig. 4). For instance, Vernadsky (65°S) was characterized by a mean summer temperature near freezing point during the period 1945–1975; mean summer temperature now reaches 0.7 °C (1985–2015 mean). Likewise, Esperanza (63°S) and Rothera (67°S) have both seen drastic summer warming, with averaged increases of 1.2 °C (from −0.2 to 1.0) and 0.6 °C (from 0.3 to 0.9), respectively (Fig. 4). Interestingly, summer temperatures at these three stations are similar to those from Bellingshausen on King George Island (62°S) and Signy Island (60°S; Fig. 4), with the latter harboring moss banks that are much thicker and older than those reported along the Antarctic Peninsula^25^.

While the effects of warmer and wetter conditions on Antarctic terrestrial ecosystems remain speculative, it is projected that many organisms and communities will be able to take advantage of, and benefit from, the ongoing changes by either expanding their range, growing their biomass, and possibly increasing trophic complexity^34^. To this effect, several studies have reported 4- to 5-fold increases in moss growth rates from moss banks over the past few decades across the maritime Antarctic region, from Elephant Island (61°S) to Lazarev Bay (69°S)^6,8,35^. Likewise, rapid increases in the range and productivity of *Deschampsia antarctica* have been reported, probably as a result of warming^5^. Other systems, however, might be vulnerable to the colonization of new taxa with better competitive abilities or representing higher trophic levels^9^. We argue that the recent resurgence of the Cape Rasmussen peatland along the Graham Coast constitutes an important new indicator for regional changes to come under warmer conditions.

In the sub-Antarctic region, peatlands are widely distributed on South Georgia (54°S), a rocky and rugged island that is home to both moss banks as well as ‘traditional’ moss-dominated and grass-dominated peatlands^36^. At Tønsberg Point, a peat deposit similar to the Rasmussen peatland was described^37^. The sequence is dominated by *Sanionia uncinata* and *Warnstorfia sarmentosum* (Wahlenb.) Hedenäs, and the peaty section dates back to approximately 4000 cal. BP. While South Georgia experiences milder conditions than the maritime Antarctic region (Fig. 4), the island can be seen as a potential analog for the west Antarctic Peninsula in terms of ecosystem development under future warming conditions. The position of the Rasmussen region in mean annual temperature and precipitation space suggests that temperatures are now approaching those recorded at more equatorward sites (Fig. 4).

One of the key reasons explaining the widespread occurrence of moss banks and the scarcity of peatlands in Antarctica might be their different surface energy balances^8^. With their saturated conditions and high water tables, peatlands dissipate energy via latent heat flux, such that their surface can be cooler than air during daytime^38^. This is not the case in dry, aerobic moss banks, which can be much warmer than surrounding air during daytime, as heat energy is produced from solar radiation^39^. In light of this evidence, the presence of a productive soligenous peatland at Cape Rasmussen is consistent with warmer summer conditions, since its microclimate may have been cooler than ambient temperatures.

Ice-free portions of the West Antarctic Peninsula amount to approximately 10,000 km^2^ ^40^. Given appropriate conditions and limited disturbance by animals, peatlands could form in this mostly barren region, as they have in South Georgia^36,37^ and across southern Patagonia^13^, and also throughout the boreal and arctic regions^41,42^. Extensive studies from northern peatlands indicate that a suitable topography, appropriate climatic conditions, a wet substrate, and the presence of plant-forming propagules are the necessary conditions for peat initiation and persistence^43^. If these conditions are met locally across the maritime Antarctic region, peatland initiation and formation could follow any of these general pathways: (1) primary formation, where peat directly forms on newly exposed, poorly drained land; (2) terrestrialization, where peatland vegetation colonizes a wet depression that had originally been accumulating inorganic material in a pond or shallow lake, or (3) paludification, where peat forms on top of mineral soils that had been developed for a long time. While peatland initiation would likely involve a hydrophile colonizing plant such as *Sanionia* sp.^44^, subsequent peatland development may involve a succession towards wet-adapted plants including *Warnstorfia sarmentosum* ^37^ or *Warnstorfia fontinaliopsis* (this study). The peat sequence could then evolve into a semi-ombrotrophic, ‘blanket mire’ dominated by *Polytrichum strictum* and/or *Chorisodontium aciphyllum*, as seen in the sub-Antarctic islands^36,44^. These dry-adapted moss species, and especially *P. strictum*, are capable of holding on to moisture for extended periods of time. While lateral expansion of these ecosystems remains hypothetical, it could occur if the peat deposits were to become saturated and that colonizing peat-forming plants were allowed to ‘swamp’ the surrounding areas, similar to what has been described for northern hemisphere systems^42,45^.

Peatlands worldwide sequester about twice as much carbon as all forests combined. While maritime Antarctic peatlands and moss banks may only account for a limited portion of the global peatland carbon store^46^, their existence provides a unique opportunity to test ideas and hypotheses about the sensitivity of peatlands to climate change. Above all, the discovery of Cape Rasmussen peatland along the Graham coast of Antarctica suggests that past and present climatic conditions have been mild enough to allow for peat initiation and subsequent development. Assuming that colonization opportunities and stabilization mechanisms would allow peat to persist in Antarctica, our results suggest that longer and warmer growing seasons in the maritime Antarctic region may lead to a peatland-rich landscape in the future.

## Methods

### Sample collection

Cape Rasmussen peatland was visited in February 2014, during a field expedition using a Zodiac boat from the Vernadsky research station. The peat core (RAS-1) was retrieved using a box corer and samples were frozen after collection.

Plant surface samples were collected around the site to identify their chemical signatures (neutral sugars and lignin phenols).

### Dating and age-depth model

All ^14^C dating was conducted on moss branches and stems (Table S1). The samples were physically cleaned and processed by routine acid-base-acid pretreatment before graphite targets were prepared. The ^14^C measurements were carried out using an accelerator mass spectrometer (AMS) at the Lawrence Livermore National Laboratory (Table S1). All ^14^C dates were calibrated using the SHCal13 data set^47^. We used median probability ages and 1σ ranges in all discussions.

### Peat geochemical, ecological, and biochemical analyses

In the laboratory, the core was cut into 1-cm-thick slices. Subsamples of 1 cm^3^ were used for estimating bulk density and organic matter content using loss-on-ignition analysis at 550 °C^48^. Subsamples of 1 cm^3^ for macrofossil analysis were sieved through a 125-μm sieve. Macrofossil remains were identified by comparing with modern reference collections and aided by illustrated flora^49^, and their abundance was estimated semi-quantitatively. Subsamples of ~5 mg dry weight were analyzed for total hydrolysable neutral sugars^50,51^; neutral sugars were separated isocratically; the seven neutral sugars measured were fucose (Fuc), rhamnose (Rha), arabinose (Ara), galactose (Gal), glucose (Glc), mannose (Man), and xylose (Xyl). Subsamples of 8–10 mg were analyzed for lignin phenols by gas chromatography/mass spectrometry following copper oxide oxidation^52^. Analyzed phenols included p-hydroxy phenols (p-hydroxybenzaldehyde, p-hydroxyacetophenone, and p-hydroxybenzoic acid), vanillyl (V) phenols (vanillin, acetovanillone, and vanillic acid), syringyl (S) phenols (syringaldehyde, acetosyringone, and syringic acid), cinnamyl (C) phenols (coumaric acid and ferulic acid), and 3,5-dihydroxy-benzoic acid.

The hemicellulosic index (HI) was calculated according to:

1 $${\rm{HI}}=({\rm{Ara}}+{\rm{Xyl}})/({\rm{Man}}+{\rm{Gal}})$$

and used unique carbohydrate compositions in peat forming plants to distinguish botanical composition. Vascular plant (*Deschampsia* and *Colobanthus*) contributions were estimated based on V and S phenols according to:

2 $${\rm{Vascular}}\,{\rm{plant}}( \% {\rm{OC}})=({{\rm{VPI}}}_{{\rm{Phenol}}}/110)\times 100$$

where VPI_Phenol_ is the vascular plant index (V + S phenols nmol﻿ mg C^−1^), and 110 nmol mg C^−1^ is the average VPI_Phenol_ in *Deschampsia* and *Colobanthus* plants. The sugar degradation index was calculated as the ratio of sugar yield in fresh peat forming plants (54 ± 12% OC) to the measured yield in samples.

## Electronic supplementary material

Table S1

## References

[1] Vaughan DG (2003). Recent rapid regional climate warming on the Antarctic Peninsula. Climatic Change.

[2] Fox AJ, Cooper APR (1998). Climate-change indicators from archival aerial photography of the Antarctic Peninsula. Annals of Glaciology.

[3] Cook AJ, Fox AJ, Vaughan DG, Ferrigno JG (2005). Retreating glacier fronts on the Antarctic Peninsula over the past half-century. Science.

[4] Cook AJ (2016). Ocean forcing of glacier retreat in the western Antarctic Peninsula. Science.

[5] Convey P, Smith RIL (2006). Responses of terrestrial Antarctic ecosystems to climate change. Plant Ecology.

[6] Amesbury, M. J. *et al*. Widespread biological response to rapid warming on the Antarctic Peninsula. *Current Biology* **27**(11), 10.1016/j.cub.2017.04.034, (2017). 10.1016/j.cub.2017.04.034 28528907

[7] Birkenmajer K, Ochyra R, Olsson IU, Stuchlik L (1985). Mid-Holocene radiocarbon-dated peat at Admirality Bay, King George Island (South Shetlands Islands, West Antarctica). Bulletin of Polish Academy of Sciences – Earth Sciences.

[8] Yu Z, Beilman D, Loisel J (2016). Transformations of landscape and peat-forming ecosystems in response to late Holocene climate change in the western Antarctic Peninsula. Geophysical Research Letters.

[9] Convey P (2006). Antarctic terrestrial ecosystems: responses to environmental change. Polarforchung.

[10] Parnikoza I (2009). Current status of the Antarctic herb tundra formation in the Central Argentine Islands. Global Change Biology.

[11] Fenton JHC, Smith RIL (1982). Distribution, composition and general characteristics of the moss banks of the maritime Antarctic. British Antarctic Survey Bulletin.

[12] Royles J, Griffiths H (2015). Climate change impacts in polar regions: Lessons from Antarctic moss bank archives. Global Change Biology.

[13] Loisel J, Yu Z (2013). Holocene peatland carbon dynamics in Patagonia. Quaternary Science Reviews.

[14] Loisel J (2014). A database and synthesis of northern peatland soil properties and Holocene carbon and nitrogen accumulation. The Holocene.

[15] Philben M, Kaiser K, Benner R (2014). Does oxygen exposure time control the extent of organic matter decomposition in peatlands?. Journal of Geophysical Research – Biogeosciences.

[16] Sarkanen, K. V. & Ludwig, C. H. Lignins: Occurrence, Formation, Structure and Reactions. Wiley, New York (1971).

[17] Philben M (2015). Temperature, oxygen, and vegetation controls on decomposition in a James Bay peatland. Global Biogeochemical Cycles.

[18] Bentley MJ (2009). Mechanisms of Holocene palaeoenvironmental change in the Antarctic Peninsula region. Holocene.

[19] Björck S, Håkansson H, Olsson S, Barnekow L, Janssens J (1993). Palaeoclimatic studies in South Shetland Islands, Antarctica, based on numerous stratigraphic variables in lake sediments. Journal of Paleolimnology.

[20] Berger A, Loutre M-F (1991). Insolation values for the climate of the last 10 million years. Quaternary Science Reviews.

[21] Renssen H, Goosse H, Fichefet T, Masson-Delmotte V, Koç N (2005). Holocene climate evolution in the high-latitude Southern Hemisphere simulated by a coupled atmospheric–sea ice–ocean–vegetation model. The Holocene.

[22] Hodgson, D. A., Doran, P. T., Roberts, D. & McMinn, A. Paleolimnological studies from the Antarctic and sub-Antarctic islands In: Pienitz, R., Douglas, M. S. V. & Smol, J. P. (Eds.) Developments in paleoenvironmental research, volume 8, long-term environmental change in Arctic and Antarctic Lakes, pp. 419-474, Springer, Dordrecht (2004).

[23] Hodgson DA (2013). Late Quaternary environmental changes in Marguerite Bay, Antarctic Peninsula, inferred from lake sediments and raised beaches. Quaternary Science Reviews.

[24] Smith RIL (1981). Types of peat and peat-forming vegetation on South Georgia. British Antarctic Survey Bulletin.

[25] Björck S (1991). Stratigraphic and paleoclimatic studies of a 5500-year-old moss bank on Elephant Island. Antarctic, Arctic and Alpine Research.

[26] Shevenell, A. E., Domack, E. W. & Kernan. G. Record of Holocene paleoclimate change along the Antarctic Peninsula: evidence from glacial marine sediments, Lallemand Fjord. In: Banks, M. R. & Brown, M. J. (Eds) Climate succession and glacial record of the Southern Hemisphere. *Proceedings of the Royal Society of Tasmania*, **130**, 55–64 (1996).

[27] Christ. AJ (2015). Late Holocene glacial advance and ice shelf growth in Barilari Bay, Graham Land, west Antarctic Peninsula. GSA Bulletin.

[28] Pudsey CJ, Evans J (2001). First survey of Antarctic sub-ice shelf sediments reveals mid-Holocene ice shelf retreat. Geology.

[29] Ciais P, Jouzel J, Petit JR, Lipenkov V, White JWC (1994). Holocene temperature variations inferred from six Antarctic ice cores. Annals of Glaciology.

[30] Masson-Delmotte V (2000). Holocene climate variability in Antarctica based on 11 ice-core isotopic records. Quaternary Research.

[31] Domack, E. W. *et al*. Marine sedimentary record of natural environmental variability and recent warming in the Antarctic Peninsula. In: Domack, E. W. (Ed.), Antarctic Peninsula Climate Variability: Historical and Paleoenvironmental Perspectives, Antarctic Research Series, vol. 79, pp. 205-222, AGU (2003).

[32] Shevenell AE, Ingalls AE, Domack EW, Kelly C (2011). Holocene Southern Ocean surface temperature variability west of the Antarctic Peninsula. Nature.

[33] Vaughan DG (2006). Recent trends in melting conditions on the Antarctic Peninsula and their implications for ice-sheet mass balance and sea level. Arctic, Antarctic, and Alpine Research.

[34] Convey P (2010). Terrestrial biodiversity in Antarctica: Recent advances and future challenges. Polar Research.

[35] Royles J (2013). Plants and soil microbes respond to recent warming on the Antarctic Peninsula. Current Biology.

[36] Van der Putten N (2009). Peat bank growth, Holocene palaeoecology and climate history of South Georgia (sub-Antarctica), based on a botanical macrofossil record. Quaternary Science Reviews.

[37] Van der Putten N, Stieperaere H, Verbruggenl C, Ochyra R (2004). Holocene palaeoecology and climate history of South Georgia (sub-Antarctica) based on a macrofossil record of bryophytes and seeds. The Holocene.

[38] Runkle BRK, Wille C, Gazovic M, Wilmking M, Kutzbach L (2014). The surface energy balance and its drivers in a boreal peatland fen of northwestern Russia. Journal of Hydrology.

[39] Robinson SA, Wasley J, Tobin AK (2003). Living on the edge – plants and global change in continental and maritime Antarctica. Global Change Biology.

[40] Brockheim, J. G. & Haus, N. W. *Distribution of Organic Carbon in the Soils of Antarctica*. In: Hartemink, A. E. & McSweeney, K. (Eds), Soil Carbon, Progress in Soil Science, chapter 37, pp. 373–380, Springer (2014).

[41] MacDonald GM (2006). Rapid early development of circumarctic peatlands and atmospheric CH_4_ and CO_2_ variations. Science.

[42] Ruppel, M., Väliranta, M., Virtanen, T. & Korhola, A. Postglacial spatiotemporal peatland initiation and lateral expansion dynamics in North America and northern Europe. *The Holocene* **23**(11), 1596–1606, 10.1177/0959683613499053 (2013).

[43] Yu, Z., Beilman, D. W. & Jones, M. C. Sensitivity of northern peatland carbon dynamics to Holocene climate change. In: Baird, A., Belyea, L., Comas, X., Reeve, A. & Slater, L. (Eds), Northern Peatlands and Carbon Cycling. *American Geophysical Union Monograph Series, Washington D.C., USA*, pp. 55–69 (2009).

[44] Smith, R. I. L. Peat forming vegetation on the Antarctic. *International Peat Society Symposium Proceedings*, pp. 58–67 (1979).

[45] Belyea, L. R. Non-linear dynamics of peatlands and potential feedbacks on the climate system. In: Baird, A., Belyea, L., Comas, X., Reeve, A. & Slater, L. (Eds), Northern Peatlands and Carbon Cycling. American Geophysical Union Monograph Series, Washington D.C., USA, pp. 5–18 (2009).

[46] Yu Z, Loisel J, Brosseau DP, Beilman DW, Hunt SJ (2010). Global peatland dynamics since the Last Glacial Maximum. Geophysical Research Letters.

[47] Hogg AG (2013). SHCal13 Southern Hemisphere calibration, 0–50,000 years cal. BP. Radiocarbon.

[48] Chambers FM, Beilman DW, Yu Z (2011). Methods for determining peat humification and for quantifying peat bulk density, organic matter and carbon content of palaeostudies of climate and peatland carbon dynamics. Mires and Peat.

[49] Ochyra, R., Smith, R. I. L. & Bednarek-Ochyra, H. The Illustrated Moss Flora of Antarctica, 704 pp., Cambridge University Press (2008).

[50] Skoog A, Benner R (1997). Aldoses in various size fractions of marine organic matter: Implications for carbon cycling. Limnology and Oceanography.

[51] Kaiser K, Benner R (2000). Determination of amino sugars in environmental samples with high salt content by high performance anion exchange chromatography and pulsed amperometric detection. Analytical Chemistry.

[52] Kaiser K, Benner R (2012). Characterization of lignin by gas chromatography and mass spectrometry using a simplified CuO oxidation method. Analytical Chemistry.

