# Supplementary material for: Peatland Ecosystem Processes in the Maritime Antarctic During Warm Climates
Source: Sci Rep. 2017 Sep 27;7:12344. doi: 10.1038/s41598-017-12479-0 (PMC5617846; doi:10.1038/s41598-017-12479-0)
Supplement: Supplementary file 1 — Table S1 [file 41598_2017_12479_MOESM1_ESM.pdf]

Peatland Ecosystem Processes in the Maritime Antarctic During Warm Climates

Julie Loisel, Zicheng Yu, David W. Beilman, Karl Kaiser, Ivan Parnikoza

Supplementary Information

Table S1. *Radiocarbon dating information.*

sample ID	Depth (cm)	Dated material	^{14}C date ± error (yr BP)	Median age[#] (cal. BP)	Lab ID
RAS-1-01	00-01[*]	moss stems	1.0379 ± 0.0033[†]	1962 or 1985 AD	CAMS-170310
RAS-1-09	09-10	moss branches	2270 ± 90	2225	CAMS-170228
RAS-1-19	19-20	moss branches	2290 ± 120	2250	CAMS-170229
RAS-1-29	29-30	moss branches	2010 ± 60	1925	CAMS-170230
RAS-1-39	39-40	moss stems	2540 ± 60	2575	CAMS-170231
RAS-1-49	49-50[**]	moss stems	2550 ± 45	2750	CAMS-170248

[*] $\delta^{13}C$ = -24.3‰ [**] $\delta^{13}C$ = -20.0‰ [#] Calibrated using the ShCal13 dataset

[†] Post-bomb date; fraction of modern radiocarbon ($F^{14}C$) is reported.
